# The LiMAx Test as Selection Criteria in Minimally Invasive Liver Surgery

**DOI:** 10.3390/jcm11113018

**Published:** 2022-05-27

**Authors:** Mirhasan Rahimli, Aristotelis Perrakis, Andrew A. Gumbs, Mihailo Andric, Sara Al-Madhi, Joerg Arend, Roland S. Croner

**Affiliations:** 1 Department of General, Visceral, Vascular and Transplant Surgery, University Hospital Magdeburg, Leipziger Str. 44, 39120 Magdeburg, Germany; aristotelis.perrakis@med.ovgu.de (A.P.); mihailo.andric@med.ovgu.de (M.A.); sara.al-madhi@med.ovgu.de (S.A.-M.); joerg.arend@med.ovgu.de (J.A.); roland.croner@med.ovgu.de (R.S.C.); 2 Department of Surgery, Centre Hospitalier Intercommunal de Poissy/Saint-Germain-en-Laye, 10 Rue du Champ Gaillard, 78300 Poissy, France; aagumbs@gmail.com

**Keywords:** LiMAx, liver function, hepatectomy, liver surgery, hepatocellular carcinoma, colorectal liver metastasis, cirrhosis, robotic surgery, laparoscopic surgery

## Abstract

Background: Liver failure is a crucial predictor for relevant morbidity and mortality after hepatic surgery. Hence, a good patient selection is mandatory. We use the LiMAx test for patient selection for major or minor liver resections in robotic and laparoscopic liver surgery and share our experience here. Patients and methods: We identified patients in the Magdeburg registry of minimally invasive liver surgery (MD-MILS) who underwent robotic or laparoscopic minor or major liver surgery and received a LiMAx test for preoperative evaluation of the liver function. This cohort was divided in two groups: patients with normal (LiMAx normal) and decreased (LiMAx decreased) liver function measured by the LiMAx test. Results: Forty patients were selected from the MD-MILS regarding the selection criteria (LiMAx normal, *n* = 22 and LiMAx decreased, *n* = 18). Significantly more major liver resections were performed in the LiMAx normal vs. the LiMAx decreased group (13 vs. 2; *p* = 0.003). Hence, the mean operation time was significantly longer in the LiMAx normal vs. the LiMAx decreased group (356.6 vs. 228.1 min; *p* = 0.003) and the intraoperative blood transfusion significantly higher in the LiMAx normal vs. the LiMAx decreased group (8 vs. 1; *p* = 0.027). There was no significant difference between the LiMAx groups regarding the length of hospital stay, intraoperative blood loss, liver surgery related morbidity or mortality, and resection margin status. Conclusion: The LiMAx test is a helpful and reliable tool to precisely determine the liver function capacity. It aids in accurate patient selection for major or minor liver resections in minimally invasive liver surgery, which consequently serves to improve patients’ safety. In this way, liver resections can be performed safely, even in patients with reduced liver function, without negatively affecting morbidity, mortality and the resection margin status, which is an important predictive oncological factor.

## 1. Introduction

Recently, an overall in-hospital mortality rate of 5.8% after liver surgery in Germany was reported. The hospital mortality was 10.4% after major liver resections [1]. Liver failure is the main reason for mortality after liver surgery. The remnant liver function after resection plays a decisive role in this situation [2]. Hence, the assessment of the pre- and postoperative liver function is a very important aspect in the perioperative management. In addition to standard laboratory blood tests for liver function and scoring systems, such as Child-Pugh or MELD scores, the LiMAx (maximum liver function capacity) test is a useful tool for the precise determination of the liver function capacity [2,3].

On the one hand, the LiMAx test may help to detect the decreased hepatocyte function which could not be determined by using standard liver function blood tests [4]. On the other hand, it can predict an improvement of liver function earlier than conventional blood parameters of liver function and clinical parameters [5]. The LiMAx test can also be used for diagnosis of chemotherapy-associated liver injury and monitoring of liver function during the transarterial chemoembolization (TACE) therapy [6,7,8]. The functional recovery of the liver after bariatric surgery can also be accurately measured by the LiMAx test [9].

For patients with chronic liver disease, the LiMAx test provides reliable results with regard to enzymatic liver function [10,11]. The LiMAx test has a prognostic value for the estimation of short-term survival of liver transplant candidates [12].

Moreover, the LiMAx test can both precisely detect the remaining liver function after liver resections and optimize the perioperative management of liver surgery. Thereby, it contributes to better patient selection [2,13,14].

We use the LiMAx test for patient selection for major or minor robotic and laparoscopic liver resections and share our experience here.

## 2. Patients and Methods

### 2.1. Patients

Patients who underwent robotic or laparoscopic minor or major liver surgery between December 2017 and May 2020 and received a LiMAx test for evaluation of the liver function as part of preoperative management were selected from the Magdeburg registry of minimally invasive liver surgery (MD-MILS). We made no selection regarding the sex, age, body mass index (BMI), number of liver lesions, tumor type and size, intrahepatic tumor localization, or previous abdominal surgery.

Our cohort consisted of 40 patients. We divided the patient cohort in two groups. The first group consisted of 22 patients with normal liver function and the second group consisted of 18 patients with impaired liver function, after evaluation with the LiMAx test.

### 2.2. Patient Selection for Major or Minor Liver Resection

Normal liver function was considered at a LiMAx cut-off value of >315 µg/h/kg, in accordance with the findings by Stockmann et al. [2]. In addition to the patient’s general condition, comorbidities, tumor location, the need for vascular reconstruction, and the presence and stage of liver cirrhosis, we used the LiMAx test for patient selection for major or minor liver resections. While the selection of patients with a normal LiMAx value for major liver resections was not a problem, we were very careful with major liver resections in patients with reduced liver function.

### 2.3. Implementation of the LiMAx Test

The LiMAx test was developed in Berlin, Germany, and first experimentally applied in 2004. This assay can be purchased from Humedics GmbH (Berlin, Germany) [14]. The LiMAx test is based on the change in CO_2_ concentration measured in the breath test, which is caused by the metabolism of the intravenously applied ^13^C-methacetin [2].

In our center, the LiMAx test is an important part of the preoperative evaluation prior to liver surgery, based on experience and reliability, especially in patients undergoing major liver resection and/or in patients with a history of liver disease, such as cirrhosis.

### 2.4. Definitions

Liver cirrhosis was determined using the Child–Pugh score on the basis of the clinical and laboratory parameters. Furthermore, it was examined histopathologically whether there were cirrhotic changes in the liver parenchyma. We defined the duration of the postoperative hospital stay as length of stay (LOS). Liver surgery related complications included posthepatectomy liver failure, intraoperative and postoperative bleeding, bile leak, bile fistula, bilioma, cholangitis, cholangiosepsis, liver abscess, and portal vein thrombosis. The 30-day mortality includes patients’ death within 30 postoperative days during hospitalization. We considered the resection of ≥3 segments as a major resection, while the resection of one or two liver segments was considered a minor resection [15]. Final diagnosis and resection margin status were determined based on histopathological examination.

### 2.5. Perioperative Management

Due to the beneficial outcomes, our center also follows the Enhanced Recovery After Surgery (ERAS) protocol in liver surgery [16,17]. Close cooperation between surgeons and anesthesiologists in the sense of the multimodal approach is very important. In addition to perioperative nutritional management; analgesia, early postoperative mobilization, where minimally invasive techniques may be advantageous; intraoperative aspects, such as preventing of intraoperative hypothermia; and balanced fluid management to maintain low central venous pressure are important factors during liver surgery [16,18].

### 2.6. Statistical Analysis

We analyzed patient characteristics and perioperative outcomes in patients with normal and decreased liver function measured by LiMAx test who underwent minor or major minimally invasive liver surgery (MILS). Moreover, anamnesis of liver disease, tumor diagnosis, and type of procedures were recorded.

The patient data were collected retrospectively. Data analysis was performed using IBM SPSS Statistics for Windows, Version 26 (IBM Corp., Armonk, NY, USA). The categorial data were presented by using the number of cases and percentages. Pearson’s chi-squared test or Fisher’s exact test were applied for the evaluation of the statistical significance. We used a Mann–Whitney U test for significance test of continuous variables. The data were presented using the mean and standard deviation (SD). Statistical significance was considered at a *p*-value of <0.05.

## 3. Results

### 3.1. Patient Demographics and Perioperative Outcomes in Patients with Normal and Decreased LiMAx Value

Table 1 shows the patient demographics and perioperative outcomes in patients with the normal and impaired liver function measured by the LiMAx test. Male patients predominated in our cohort, which consisted of 25 (62.5%) male and 15 (37.5%) female patients. Our patients were 66.9 (SD 10.5) years old on average. The mean BMI was 27.9 (SD 5.0) kg/m^2^, which was statistically significant higher in the decreased LiMAx group (*p* = 0.005). The mean LiMAx value was in the normal LiMAx groups 466.2 (SD 114.8) and in the decreased LiMAx group 234.9 (SD 56.8) µg/h/kg, respectively, with statistical significance of *p* < 0.001. Two patients (9.1%) in the normal, and ten patients (55.6%) in the decreased LiMAx group were recorded with liver cirrhosis.

The normal LiMAx group showed a significantly longer operation time than decreased LiMAx group (356.6 (SD 148.6) min vs. 228.1 (SD 107.2) min; *p* = 0.003). In the normal LiMAx group, the intraoperative blood loss was higher than in the reduced LiMAx group (495.5 vs. 380.6 mL). However, this difference was not statistically significant. Intraoperative blood transfusion was required significantly more frequently in the normal LiMAx group than in the decreased LiMAx group (8 (36.4%) vs. 1 (5.6%); *p* = 0.027). This discrepancy in significance could be explained by the different types of variables in blood loss and blood transfusion and different indications for blood transfusion. For example, the indication for blood transfusion could be given rather in elderly patients or patients with comorbidities such as heart disease, even if the blood loss does not appear to be significantly high.

There was no significant difference between two groups in terms of length of hospital stay, liver surgery related morbidity, or mortality.

One patient died on the 5th postoperative day after major liver resection for a hepatocellular carcinoma (HCC) due to pulmonary embolism. That was the only in-hospital mortality (2.5%) in our cohort.

We performed 13 major resections (59.1%) in the normal and 2 major resections (11.1%) in the decreased LiMAx group (*p* = 0.003).

More than half of our patients (52.5%) showed relevant intra-abdominal adhesions caused by previous abdominal surgery. We recorded 34 (85.0%) malignant and 6 (15.0%) non-malignant cases in our cohort. There were no significant differences between the groups with regard to the distribution of intra-abdominal adhesions and tumor dignity.

The R0 resection was achieved in 31 (91.2%) patients. Three patients showed microscopically positive resection margins; one of them was in the normal, and two patients in the decreased LiMAx group (*p* = 0.591).

### 3.2. Cirrhosis Anamnesis, Liver Disease and Diagnosis

We recorded ten patients (25.0%) with Child A and two patients (5.0%) with Child B liver cirrhosis in our cohort. There was no patient with Child C liver cirrhosis in this study.

The underlying liver diseases were hepatic steatosis in fourteen cases (35.0%), hepatitis B infection in two cases (5.0%) and hepatitis C infection in one case (2.5%).

The most common malignant diagnosis was hepatocellular carcinoma (HCC), with 17 cases (42.5%). The remaining malignant diagnoses included colorectal liver metastases, cholangiocarcinoma (CCA) and combined cases of HCC and CCA. The non-malignant diagnoses were liver hemangioma, hepatic adenoma, and inflammatory tumor. These findings are illustrated in Table 2.

### 3.3. Type of Liver Resections

Table 3 shows the procedures performed. We carried out eight (20.0%) right, and four (10.0%) left hemihepatectomies. The remaining major liver resections were resections of three (*n* = 2; 5%) and four liver segments (*n* = 1; 2.5%).

Left lateral liver resection was the most common minor procedure (*n* = 10; 25.0%). The remaining minor resections were the anatomical and atypical resections of one liver segment, bisegmentectomies, and atypical resection of two liver segments.

### 3.4. Characteristics of Malignant Cases

A total of 34 malignant cases were identified in our patient cohort. Twenty-four of these were primary liver malignancies. Table 4 illustrates the histopathological characteristics of primary liver malignancies. Seventeen HCC, five CCA, and two mixed cases of HCC and CCA were detected. Twelve pT2, nine pT1, and three pT3 tumors were identified. Most tumors showed G2 grading (*n* = 15; 62.5%). A histologically positive resection margin was found in one case with HCC and one case with CCA. The mean diameter of the largest lesion was 44.3 (SD 28.1) mm.

Ten patients with colorectal liver metastases underwent minimally invasive liver surgery. Five of them had rectal cancer and the remaining five patients had colon cancer. Table 5 shows characteristics of cases with colorectal liver metastases. The colorectal liver metastases occurred metachronous in five cases and synchronous in five cases. All patients had received chemotherapy or radiochemotherapy at the time of index liver surgery. Microscopically positive resection margins were found in only one case. The mean diameter of the largest lesion was 42.8 (SD 20.0) mm. The metastases showed a tumor regression grade III-V according to Rubbia-Brandt et al. [19].

## 4. Discussion

A multicenter, randomized clinical trial reported by Stockmann et al. showed that the postoperative management after liver resection can be improved by using the LiMAx test perioperatively. This can lead to a reduction in the rate of severe postoperative complications [14]. Using a LiMAx decision tree algorithm for planned liver resections, the postoperative morbidity after hepatectomy could be reduced significantly. Depending on the liver function and the extent of the intended liver resection, a decision should be made about the further treatment steps [2]. This concept was verified in a later study including 1170 liver resections. The results show that the use of the LiMAx algorithm reduced postoperative liver failure and postoperative liver failure-related mortality [13].

According to the prospective study by Kaffarnik et al., the liver dysfunction after major abdominal surgery can be accurately determined using the LiMAx test, whereby there was no difference between laparoscopic and open procedures regarding the hepatic dysfunction [20].

In most cases, patients in our department, indicated for liver resection, undergo a LiMAx test in the preoperative setting in order to assess their liver function. All patients with a normal LiMAx value (>315 µg/h/kg) are eligible for all forms of liver resection, including major liver surgery. In patients with a reduced LiMAx value (<315 µg/h/kg), the extent of the liver resection must be carefully considered. Factors such as the patient’s general condition, comorbidities, tumor location, and the need for vascular reconstruction play a relevant role. After considering all of the parameters, it must be thoroughly evaluated whether a major liver resection can be performed in this critical patient population with restricted liver function. If not, then a parenchymal-sparing procedure could be selected, e.g., minor resections, atypical liver resections, wedge resections, or local excision of the tumors. If there are multiple liver lesions, a hybrid procedure, for example, combination of surgical and radiological interventional procedures, could be used. The “surgically well accessible” lesions can be resected, and those that would require the sacrifice of a large volume of the liver parenchyma if surgically removed could be treated interventionally, e.g., radiofrequency ablation, laser ablation, etc.

Ultimately, we selected our patients for major or minor liver resections based on liver function measured by LiMAx test. Accordingly, we had a significantly higher rate of major liver resections in the normal LiMAx group (*p* = 0.003), which explains the significantly longer operation time in this group. However, there was no significant difference between the normal and decreased LiMAx groups regarding the intraoperative blood loss, length of hospital stay, liver surgery related morbidity, 30-day mortality and R status. The patient selection using the LiMAx test allows us to perform liver resections safely even in patients with reduced liver function without negatively affecting the postoperative results including the morbidity, mortality and resection margin status.

The limitations of our study are the small patient cohort and the retrospective nature. Studies with larger sample size and randomization are required to substantiate the results so far.

## 5. Conclusions

The LiMAx test is a very helpful and reliable tool to precisely determine the liver function capacity. We shared our experiences with the LiMAx test in patient selection for minor and major minimally invasive liver surgery. After careful patient selection, liver resections can be carried out safely, even in patients with reduced liver function, without negatively affecting morbidity, mortality and the resection margin status, which is an important predictive oncological factor.

## Figures and Tables

**Table 1:** Patient demographics and perioperative outcomes in patients with normal and decreased LiMAx value who underwent minor or major minimally invasive liver surgery (MILS).

		Normal LiMAx Value (>315 µg/h/kg) *n* (% or SD)	Decreased LiMAx Value (≤315 µg/h/kg) *n* (% or SD)	*p*-Value	Total *n* (% or SD)
Total		22 (55.0)	18 (45.0)		40 (100.0)
Sex	male	12 (54.5)	13 (72.2)	0.332	25 (62.5)
	female	10 (45.5)	5 (27.8)		15 (37.5)
Age; years		64.0 (12.0)	70.6 (7.2)	0.058	66.9 (10.5)
BMI; kg/m^2^		25.9 (4.0)	30.4 (5.2)	**0.005**	27.9 (5.0)
LiMAx value; µg/h/kg		466.2 (114.8)	234.9 (56.8)	**<0.001**	362.1 (148.6)
Liver cirrhosis		2 (9.1)	10 (55.6)	**0.002**	12 (30.0)
Liver cirrhosis	None	20 (90.9)	8 (44.4)	**0.005**	28 (70.0)
	Child A	2 (9.1)	8 (44.4)		10 (25.0)
	Child B	0 (0.0)	2 (11.1)		2 (5.0)
Operation time; min		356.6 (148.6)	228.1 (107.2)	**0.003**	298.8 (145.2)
IBL; mL		495.5 (470.0)	380.6 (352.8)	0.459	443.8 (420.2)
Intraoperative blood transfusion		8 (36.4)	1 (5.6)	**0.027**	9 (22.5)
LOS; days		15.6 (14.2)	11.5 (9.8)	0.338	13.8 (12.4)
Liver surgery related morbidity		4 (18.2)	1 (5.6)	0.355	5 (12.5)
30-day mortality		1 (4.6)	0 (0.0)	1.000	1 (2.5)
Operation technique	laparoscopic	4 (18.2)	12 (66.7)	**0.003**	16 (40.0)
	robotic	18 (81.8)	6 (33.3)		24 (60.0)
Extent of resection	major	13 (59.1)	2 (11.1)	**0.003**	15 (37.5)
	minor	9 (40.9)	16 (88.9)		25 (62.5)
Previous abdominal surgery		13 (59.1)	8 (44.4)	0.525	21 (52.5)
Tumor dignity	malignant	18 (81.8)	16 (88.9)	0.673	34 (85.0)
	benign	4 (18.2)	2 (11.1)		6 (15.0)
R status in malignant cases	R0	17 (94.4)	14 (87.5)	0.591	31 (91.2)
	R1	1 (5.6)	2 (12.5)		3 (8.8)

BMI = body mass index, IBL = intraoperative blood loss, LOS = length of stay, SD = standard deviation. Significant values (*p* < 0.05) marked in bold.

**Table 2:** Cirrhosis anamnesis, liver disease, and diagnosis.

		Total *n* (%)
Total number of patients		40 (100.0)
Liver cirrhosis	None	28 (70.0)
	Child A	10 (25.0)
	Child B	2 (5.0)
Liver Disease	Hepatic steatosis	14 (35.0)
	Hepatitis B	2 (5.0)
	Hepatitis C	1 (2.5)
Type of liver lesion	HCC	17 (42.5)
	Colorectal metastases	10 (25.0)
	CCA	5 (12.5)
	HCC + CCA	2 (5.0)
	Liver hemangioma	3 (7.5)
	Hepatic adenoma	2 (5.0)
	Inflammatory tumor	1 (2.5)

CCA = cholangiocarcinoma, HCC = hepatocellular carcinoma.

**Table 3:** Procedures in patients who underwent minor or major minimally invasive liver surgery (MILS).

		Total *n* (%)
Major resections		
	Right hemihepatectomy	8 (20.0)
	Left hemihepatectomy	4 (10.0)
	Resection of 3 segments	2 (5.0)
	Resection of 4 segments	1 (2.5)
Minor resections		
	Left lateral liver resection	10 (25.0)
	Anatomical one segment resection	7 (17.5)
	Bisegmentectomy	4 (10.0)
	Atypical one segment resection	3 (7.5)
	Atypical resection of two segments	1 (2.5)
Total		40 (100.0)

**Table 4:** Histopathological characteristics of primary liver malignancies.

Liver Malignancy	T	V	L	Pn	G	R	Largest Lesion (mm)
HCC	pT3	V0	L0	Pn0	G1	R0	105
HCC	pT3	V1	L0	-	G2	R1	60
HCC	pT2	V1	L0	Pn0	G1	R0	62
HCC	pT2	V1	L0	Pn0	G2	R0	30
HCC	pT2	V1	-	-	G2	R0	20
HCC	pT3	V1	L0	Pn0	G2	R0	53
HCC	pT2	V0	L0	Pn0	G2	R0	32
HCC	pT1	V0	L0	Pn0	G1	R0	18
HCC	pT2	-	-	-	G2	R0	6
HCC	pT2	V1	-	-	G3	R0	43
HCC	pT1	V0	L0	Pn0	G2	R0	32
HCC	pT2	V1	L0	-	G3	R0	30
HCC	pT2	V1	L0	Pn0	G2	R0	42
HCC	pT2	V0	L0	Pn0	G2	R0	40
HCC	pT1	V0	L0	Pn0	G1	R0	30
HCC	pT1	V1	L0	Pn0	G2	R0	20
HCC	pT1	V0	L0	Pn0	G1	R0	16
CCA	pT2	V1	L1	Pn1	G2	R0	90
CCA	pT2	V1	L0	Pn1	G2	R0	75
CCA	pT2	V1	L1	Pn0	G3	R0	80
CCA	pT1	-	-	-	G2	R1	12
CCA	pT1	V0	L0	Pn0	G2	R0	30
HCC + CCA	pT1	V0	L0	Pn0	G2	R0	100
HCC + CCA	pT1	V0	L0	Pn0	-	R0	36

CCA = cholangiocarcinoma, HCC = hepatocellular carcinoma; T = T stage, V = invasion into vein, L = invasion into lymphatic vessels, P = perineural invasion, G = tumor grading, R = resection margin status.

**Table 5:** Characteristics of colorectal liver metastases.

Patient No.	Primarius	Time of Occurence	RCT Oder CT Prior to Index Liver Surgery	R Status	Largest Lesion (mm)	TRG
1.	Rectum cancer	metachron	yes	R0	18	-
2.	Rectum cancer	metachron	yes	R0	54	-
3.	Colon cancer	synchron	yes	R0	60	III
4.	Colon cancer	synchron	yes	R0	45	IV
5.	Rectum cancer	synchron	yes	R0	38	V
6.	Colon cancer	synchron	yes	R0	18	III
7.	Rectum cancer	synchron	yes	R0	34	IV
8.	Colon cancer	metachron	yes	R0	36	-
9.	Colon cancer	metachron	yes	R1	40	IV
10.	Rectum cancer	metachron	yes	R0	85	-

CT = chemotherapy, RCT = radiochemotherapy, TRG = tumor regression grade according to Rubbia-Brandt et al. [19].

## Data Availability

The datasets used and/or analyzed during the current study are available from the corresponding author on reasonable request.
